# Prediction of exercise-induced desaturation in COPD patients without resting hypoxemia: a retrospective study

**DOI:** 10.1186/s12890-022-02174-w

**Published:** 2022-11-08

**Authors:** Lulu Yang, Minghui Shi, Xuanming Situ, Jiaze He, Shiwei Qumu, Ting Yang

**Affiliations:** 1 Department of Pulmonary and Critical Care Medicine, China-Japan Friendship Hospital, Yinghuayuan East Street, Chaoyang District, Beijing, 100029 China; 2 National Center for Respiratory Medicine, Beijing, 100029 China; 3 National Clinical Research Center for Respiratory Diseases, Beijing, 100029 China; 4 Fangzhuang Community Health Service Center, Capital Medical University, Beijing, 100078 China; 5 Capital Medical University, Beijing, 100069 China; 6 Department of Rehabilitation Medicine, China-Japan Friendship Hospital, Beijing, 100029 China; 7 Institute of Respiratory Medicine, Chinese Academy of Medical Sciences, Beijing, 100029 China

**Keywords:** Chronic obstructive pulmonary disease, 6-min walk test, Exercise-induced desaturation, COPD assessment test, CT defined, Emphysema

## Abstract

**Background:**

There is no universally accepted criterion for assessing exercise-induced desaturation (EID). The purpose of this study is to compare the two methods regularly used for determining EID in COPD patients, as well as to explore the risk factors and predictors related to EID.

**Methods:**

The 6MWT was performed with continuous SpO_2_ monitoring on patients with stable COPD. Using two methods (method A: “SpO_2rest_–SpO_2min_ ≥ 4% and/or SpO_2min_ < 90%”, method B: “SpO_2rest_–SpO_2end_ ≥ 4% and/or SpO_2end_ < 90%”) as EID determination criteria to assess the incidence of EID. The differences and consistency of the two methods are compared. Moreover, we collected data through the pulmonary function test, mMRC dyspnea score, COPD assessment test, BODE index and CT-defined emphysema. Univariate and multivariate logistic regression analyses were used to identify factors affecting the EID. For the parameters that predict EID in 6MWT, a receiver operating characteristic (ROC) curve analysis was employed.

**Results:**

The analysis included 124 patients. The overall incidence of EID was 62.1% by using method A as the criterion and 51.6% by method B. All of the EID patients found by method B were included in the EID patients identified by method A, as well as 13 new-EID patients. The difference in diagnostic outcomes between the two approaches was not statistically significant (*P* > 0.05), but they were in excellent agreement (Kappa = 0.807, *P* = 0.001). Logistic regression analyses found that D_L_CO SB% pred, D_L_CO/VA% pred, CAT score, mean density, PD15, emphysema volume and %LAA were significant determinants of the EID. For predicting EID, the ROC analysis produced AUC and cutoffs of 0.689 and 50.45% (D_L_CO SB% pred), 0.707 and 75.0% (D_L_CO/VA% pred), 0.727 and 15 points (CAT score), 0.691 and − 955.00HU (PD15), 0.671 and − 856.46HU (mean density), 0.668 and 338.14 ml (emphysema volume) and 0.656 and 7.63% (%LAA), respectively.

**Conclusions:**

Two methods evaluating EID in this research are in a good agreement, method A can find more EID patients by focusing on SpO_2min._ When conditions are constrained, it is also sufficient to assess EID in COPD patients by method B. In terms of the predictors of EID, D_L_CO SB% pred, D_L_CO/VA% pred, CAT score and CT-defined emphysema are all statistically significant test variables to determine EID.

## Introduction

Chronic obstructive pulmonary disease (COPD) is a chronic respiratory disease characterized by irreversible persistent airflow limitation, with high prevalence, mortality and disease burden [1]. Exercise-induced desaturation (EID) is a term used to describe patients with COPD who do not have hypoxemia at rest but have a desaturation during exercise. In contrast to the classical cardiopulmonary exercise test, the 6-min walk test (6MWT) does not require large professional medical equipment, is simple to perform, is acceptable to most patients in terms of exercise intensity, and is closer to patients’ daily lives in terms of exercise form, and is now more widely used to monitor EID than the classical cardiopulmonary exercise test [2].

Different clinical investigations utilize different criteria to determine EID because there are no common standards or definitions. The following are commonly used: (1) the difference between the oxygen saturation (SpO_2_) at the beginning of the test (SpO_2rest_) and the SpO_2_ at the end of the test (SpO_2end_) (SpO_2rest_–SpO_2end_) ≥ 4% and/or the SpO_2end_ < 90% [3–7], (2) the difference between the SpO_2rest_ and the minimum SpO_2_ during the test (SpO_2min_) (SpO_2rest_–SpO_2min_) ≥ 4% and/or the SpO_2min_ < 90% [8], (3) SpO_2min_ ≤ 88% [9], (4) SpO_2end_ ≤ 88% [7, 10]. A study compared “SpO_2end_ ≤ 88%” and “SpO_2rest_–SpO_2end_ ≥ 4% and/or the SpO_2end_ < 90%” in 507 patients with COPD, the incidence of EID was 5.1% (26/507) by the former method and 13% (66/507) by the latter. Although the former found a low incidence of EID in COPD patients, it had a stronger prognostic value for long-term mortality than the latter after 162 months of follow-up [7]. It is certain that EID is prevalent in COPD patients, regardless of the determination method. However, few studies have focused on the SpO_2min_ during 6MWT [8, 9], potentially misclassifying some patients who desaturate during the 6MWT but do not desaturate at the conclusion as “non-EID patients”. Therefore, continuous SpO_2_ monitoring is required during the 6MWT to record the SpO_2min_ during the trial.

Emphysema plays a role in the onset and course of EID [10]. CT-defined emphysema is more sensitive and repeatable than subjective eye scoring systems, and it also allows for emphysema spatial distribution and localisation. Most studies used − 950 Hounsfield units (HU) as the ideal threshold for diagnosis of CT defined emphysema [11–13]. The most widely used indicators for evaluating emphysema were lung volumes with X-ray attenuation values below − 950 HU, percentage low attenuation regions (% LAA), the 15th percentile point of density (PD 15), and the mean density of the lung [13–15]. Because it is less affected by picture noise, PD15 is more commonly utilized in longitudinal studies of emphysema [14, 15]. Studies shows that %LAA is independently associated with EID [16, 17]. According to the findings, each 1% increase in percent LAA raises the relative risk of EID in the 6MWT by 10% and the relative risk of EID recurrence by 20%, each 1% increase in %LAA increases the relative risk of EID in 6MWT by 10% and the relative risk of reoccurrence of EID by 20% [18]. Few studies, however, have looked at the relative predictive value and threshold of the mean density, PD15, emphysema volume, and %LLA for EID at the same time.

In patients with COPD, EID is linked to lower quality of life, decreased exercise tolerance, higher readmission rates for acute exacerbations, and increased morbidity and mortality [3, 19–21]. On the one hand, identifying whether markers indicate EID occurrences may assist physicians in assisting this group of patients in conducting the 6MWT with oxygen, which may help them avoid terminating the trial when the patients' blood oxygen levels are too low (less than 85% or 80%) [22, 23]. On the other hand, it can reflect the patient’s most realistic exercise capacity while also reducing the danger of hypoxia-related exercise.

Therefore, the primary goal of this research was to answer the following three questions: (1) Obtain SpO_2min_ by continuously measuring SpO_2_ during 6MWT. Using two methods (method A: “SpO_2rest_–SpO_2min_ ≥ 4% and/or SpO_2min_ < 90%”, method B: “SpO_2rest_–SpO_2end_ ≥ 4% and/or SpO_2end_ < 90%”) as EID determination criteria to assess the incidence of EID in patients with COPD during 6MWT. Comparing the differences and consistency between the two methods. (2) What are the clinical features of patients with EID who were diagnosed using the former technique but not the latter? (3) To investigate the predictive value of different indicators for EID by comparing questionnaire scores, pulmonary function and degree of emphysema in patients with or without EID. To find more sensitive predictors of EID and timely detection of EID events in COPD.

## Methods

### Patients

A total of 124 consecutive patients with COPD who met the inclusion criterion and did not meet the exclusion criteria were enrolled in the study between January 2019 and December 2021 in the Department of Respiratory and Critical Care Medicine of the China-Japan Friendship Hospital. The inclusion criterion was a diagnosis of stable COPD according to the Global Initiative for Chronic Obstructive Lung Disease (GOLD), as updated in 2020. The exclusion criteria were as follows: (1) SpO_2rest_ < 94%; (2) unstable angina pectoris or myocardial infarction; (3) recently diagnosed pulmonary embolism and severe pulmonary hypertension in 1 month; (4) resting heart rate > 120 beats/min; (5) systolic blood pressure > 180 mmHg (1 mmHg = 0.133 kPa) and/or diastolic blood pressure > 100 mmHg; (6) malignant arrhythmia; (6) severe valvular disease; (7) limitations on walking including predominant neurological or musculoskeletal limitation; (8) Combined with a malignant tumor [22].

### 6MWT

The 6MWT was performed on a 30 m, flat, straight indoor walking course, supervised by at least two experienced investigators following the ATS guidelines [22]. Continuous measurements of SpO_2_ were performed using a finger pulse oximeter that transmits data in real time via Bluetooth mentioned from 1 min before the beginning of the 6MWT to the fourth minute after the 6MWT. The 6MWD, SpO_2rest_, SpO_2end_, SpO_2min_ was measured, then calculate SpO_2rest_–SpO_2end_ and SpO_2rest_–SpO_2min_. Use two methods: A: “SpO_2rest_–SpO_2min_ ≥ 4% and/or SpO_2min_ < 90%” and B: “SpO_2rest_–SpO_2end_ ≥ 4% and/or SpO_2end_ < 90%” as EID determination criteria to assess the incidence of EID in 6MWT. Patients determined to be EID by method A were defined as “EID patients”, patients determined to be EID by method A but not by method B were defined as “new-EID patients”, patients determined to be EID by both methods were defined as “original-EID patients”, and patients determined to be non-EID by both methods were defined as “non-EID patients”.

### Pulmonary function

Pulmonary function was performed by well-trained hospital staff according to the American Thoracic Society (ATS) and the European Respiratory Society (ERS) guidelines using a Jaeger^®^ MasterScreen system (Jaeger^®^, Viasys Healthcare GmbH, Hochberg, Germany) [24].

### Questionnaires

Dyspnea was measured using the modified Medical Research Council (mMRC) dyspnea score [25], health-related quality of life and symptom burden of COPD was measured using the COPD Assessment Test (CAT) [26]. The BODE index was calculated according to BMI (B), airflow obstruction (O), dyspnea (D) and exercise ability (E) [27].

### CT defined emphysema

**Fig. 1:**
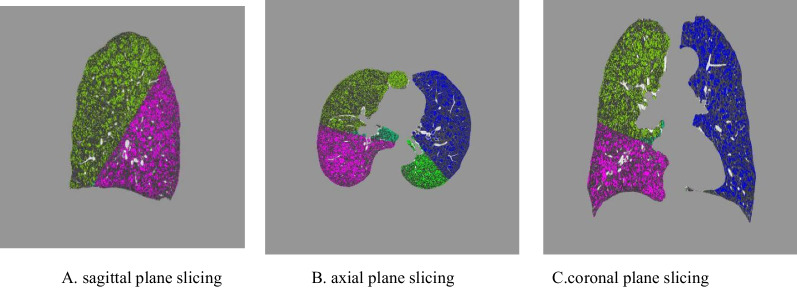
CT defined emphysema reconstruction diagram

All patients underwent CT scans using GE Healthcare multidetector-row CT scanner. The scans were done at suspended full inspiration. Exposure settings were 120 kVp and 40 mAs, images were reconstructed at 1.0 or 5.0 mm contiguous slices. The CT scans were analyzed using FACT Medical Imaging System 1.2.0 software. Briefly, the lungs were segmented from the thorax wall, heart, and main pulmonary vessels, followed by segmentation of the individual lobes. A density of less than − 950HU is used as the threshold for emphysema reconstruction [11–13], the lung volumes with X-ray attenuation values below − 950 HU, %LAA, the PD 15 and the mean density of the lung were calculated to assess the degree of emphysema [13–15] (Fig. 1).

### Statistical analysis

The statistical analyses were performed with SPSS statistics software (version 23.0; SPSS Inc., Chicago, IL). Categorical variables were expressed as compositional ratios, and the chi-square test was used. We used the Shapiro–Wilk test for the analysis of the normality of the data; results were described as mean and standard deviation or median and interquartile range (IQR) according to the data distribution. To determine whether differences were present between groups, selected characteristics were assessed using a chi-square test or independent t-test. Kappa verified the agreement of the two determination methods. Univariate and multivariate logistic regression analyses were used to identify factors affecting the EID. Then a receiver operating characteristic (ROC) curve analysis and the area under the curve (AUC) were used to determine the optimal cutoff value for the factors predicting EID. *P* values < 0.05 were considered statistically significant.

## Results

### Clinical characteristics

This study included 101 men and 23 women with stable COPD; among them, 37 patients underwent CT in 5 mm and 87 patients in 1 mm. The severity of airway limitation was classified as GOLD stage: mild (n = 24), moderate (n = 43), severe (n = 33) and very severe (n = 24). The mean SpO_2rest_–SpO_2min_ in this study was 7.05 ± 6.79%, and the mean SpO_2rest_–SpO_2end_ was 5.28% ± 6.66%. By using method A as the criterion, the overall incidence of EID was 62.1% (EID patients, n = 77) and 51.6% (original-EID patients, n = 64) by method B. All of the EID patients found by method B were included in the EID patients identified by method A, as well as 13 new EID patients. There was no statistically significant difference between the diagnostic outcomes of the two procedures (*P* > 0.05, Table 1). The diagnostic results of the two approaches were in good agreement in terms of consistency (Kappa = 0.807, *P* < 0.001). Table 2 lists the features of these individuals in various groups. In terms of age, height, body mass, BMI, FVC, FEV1, FVC% pred, FEV1%pred, and SpO_2rest_, no statistically significant changes were identified between EID and non-EID patients(*P* > 0.05). However, there were statistically significant differences in gender, 6MWD, SpO_2min_, SpO_2end_, SpO_2rest_–SpO_2min_, SpO_2rest_–SpO_2end_, diffusion function, questionnaire scores and degree of emphysema (*P* < 0.05, Table 2). Only the degree of SpO_2_ change in 6MWT differed statistically significant between the new-EID patients (n = 13) who were not detected by method B but detected by method A and original-EID patients (n = 64) who were detected by both methods (Table 2).

**Table 1:** Comparison of the incidence of EID by GOLD classification under different determination methods

Determination methods	GOLD classification [n (%)]				
	Stage I (n = 24)	Stage II (n = 43)	Stage III (n = 33)	Stage IV (n = 24)	Total (n = 124)
Method A	12 (50.0)	28 (65.1)	20 (60.6)	17 (70.8)	77 (62.1)
Method B	10 (41.7)	24 (55.8)	15(45.5)	15 (62.5)	64 (51.6)
χ^2^	0.336	0.778	1.521	0.375	2.778
*P*	0.562	0.378	0.218	0.540	0.096

*EID* Exercise-induced desatuation; *method A* SpO_2rest_–SpO_2min_ ≥ 4% and/or SpO_2min_ < 90%; *method B* SpO_2rest_–SpO_2end_ ≥ 4% and/or SpO_2end_ < 90%; *SpO*_*2rest*_ resting blood oxygen saturation at the beginning of the test; *SpO*_*2min*_ minimum blood oxygen saturation during the test; *SpO*_*2end*_ blood oxygen saturation at the end of the test

**Table 2:** Comparison of relevant indicators between different groups

Variables	new-EID patients (*n* = 13)	original-EID patients (*n* = 64)	*P*	EID patients (*n* = 77)	non-EID patients (*n* = 47)	*P*	P*
Male (n, %)	11 (84.62%)	56 (87.5%)	0.778	67 (87.01%)	34 (72.34%)	0.041	
Age (y)	63.85 ± 9.08	64.44 ± 10.10	0.846	64.34 ± 9.88	63.21 ± 11.30	0.562	0.853
Height (m)	1.67 (1.58, 1.76)	1.64 (1.62, 1.72)	0.623	1.64 ± 20.57	1.66 ± 7.83	0.544	0.112
Weight (kg)	62.00 (57.00, 72.00)	64.25 (55.73, 72.30)	0.739	65.46 ± 13.18	64.06 ± 11.68	0.550	0.983
BMI (kg/m^2^)	21.51 (20.84, 24.45)	23.09 (20.58, 25.13)	0.545	23.14 ± 4.24	23.94 ± 3.53	0.280	0.291
GOLD stage (I: II: III: IV)	2:4:5:2	10:24:15:15	0.724	12:28:20:17	12:15:13:7	0.481	0.879
*Baseline 6MWT*							
6MWD (m)	397.85 ± 112.56	415.78 ± 92.18	0.540	411.45 ± 94.54	464.85 ± 100.28	0.003	0.042
SpO_2rest_ (%)	96.00 (95.00, 97.00)	96.00 (95.00, 97.00)	0.699	96.00 (94.00, 97.00)	95.00 (94.00, 97.00)	0.329	0.576
SpO_2min_ (%)	91.00 (88.50, 93.50)	83.00 (80.00, 88.70)	< 0.001	87.00 (80.00, 90.00)	94.00 (94.00, 95.00)	< 0.001	< 0.001
SpO_2end_ (%)	94.00 (93.50, 96.00)	86.00 (84.00, 91.00)	< 0.001	88.00 (84.00, 92.00)	95.00 (94.00, 97.00)	< 0.001	0.027
SpO_2rest_–SpO_2min_ (%)	4.00 (4.00, 8.00)	10.50 (7.00, 16.80)	< 0.001	9.00 (6.00, 15.00)	2.00 (0.00, 3.00)	< 0.001	< 0.001
SpO_2rest_–SpO_2end_ (%)	2.00 (0.00, 3.00)	8.50 (6.00, 12.00)	< 0.001	6.00 (4.00, 11.00)	0.00 (-2.00, 2.00)	< 0.001	0.005
*Lung function index*							
FVC (L)	3.07 ± 0.78	2.98 ± 0.83	0.751	3.00 ± 0.82	2.88 ± 0.91	0.454	0.505
FVC% pred (%)	94.69 ± 24.48	87.04 ± 22.83	0.295	88.24 ± 23.10	88.70 ± 21.46	0.915	0.406
FEV_1_ (L)	1.47 (0.76, 2.60)	1.35 (0.84, 1.87)	0.786	1.43 ± 0.68	1.42 ± 0.59	0.953	0.650
FEV_1_% pred (%)	61.72 (34.15, 96.00)	51.57 (29.76, 70.54)	0.663	53.05 ± 25.96	57.29 ± 22.89	0.202	0.963
D_L_CO SB (mmol/min/kPa)	5.01 ± 1.85	4.63 ± 1.74	0.497	4.69 ± 1.76	5.72 ± 1.52	0.002	0.117
D_L_COSB% pred (%)	63.79 ± 22.09	56.37 ± 20.31	0.240	58.76 ± 22.29	72.66 ± 22.51	0.001	0.096
D_L_CO/VA (mmol/min/kPa/L)	1.14 ± 0.32	1.00 ± 0.34	0.189	1.02 ± 0.34	1.31 ± 0.37	< 0.001	0.145
D_L_CO/VA% pred (%)	84.88 ± 22.24	75.06 ± 22.98	0.162	75.10 ± 23.15	93.13 ± 23.17	< 0.001	0.176
*Questionnaires*							
mMRC dyspnea scale	2 (1, 3)	2 (1, 3)	0.709	2 (1, 3)	2 (1, 2)	0.019	0.049
CAT score	19 (11, 26)	20 (15, 25)	0.978	20 (14.5, 25)	12 (7, 18)	< 0.001	0.009
BODE index	2 (1.5, 5)	3 (1, 5)	0.863	3 (1.5, 5)	2 (1, 4)	0.02	0.113
*CT defined emphysema*							
mean density (HU)	− 848.20 (− 882.06, − 822.97)	− 843.44 (− 868.22, − 813.52)	0.251	− 860.10 ± 36.15	− 836.41 ± 40.18	0.028	0.157
PD15 (HU)	− 946.82 ± 32.69	− 961.40 ± 25.08	0.094	− 959.23 ± 26.61	− 946.43 ± 26.21	0.014	0.967
emphysema volume (ml)	159.84 (59.93, 1930.92)	579.56 (207.13, 1458.54)	0.245	578.50 (178.75, 1473.75)	196.66 (77.26, 694.12)	0.003	0.350
%LAA (%)	3.77 (1.36, 33.06)	12.61 (4.47, 21.79)	0.218	11.00 (4.00, 22.00)	4.16 (1.88, 12.01)	0.005	0.280
mean density of right lung (HU)	− 849.18 (− 877.59, − 822.87)	− 843.42 (− 872.72, − 822.36)	0.391	− 850.50 (− 875.50, − 824.50)	835.57 (− 855.24,− 807.53)	0.066	0.159
PD15 of right lung (HU)	− 946.09 ± 31.73	− 960.14 ± 25.09	0.472	− 958.05 ± 26.42	− 945.10 ± 27.96	0.014	0.919
emphysema volume of right lung (ml)	92.82 (37.81, 914.22)	345.80 (92.20, 702.27)	0.251	311.50 (86.75, 798.5)	98.9 (41.26, 425.47)	0.004	0.471
%LAA of right lung (%)	4.28 (1.54, 29.21)	12.87 (4.35, 20.61)	0.218	12.50 (4.00, 22.00)	3.49 (1.63, 12.47)	0.004	0.388
mean density of left lung (HU)	− 847.17 (− 887.17, − 822.11)	− 850.85 (− 869.02, − 919.80)	0.399	− 851.00 (− 875.50, − 822.00)	− 832.31 (− 858.65, − 797.52)	0.012	0.434
PD15 of left lung (HU)	− 940.00 (− 986.00, − 920.00)	− 964.50 (− 980.00, − 942.50)	0.210	− 958.09 ± 28.97	− 946.67 ± 28.19	0.414	0.937
emphysema volume of left lung (ml)	69.02 (22.11, 1016.70)	306.92 (117.48, 797.10)	0.335	285.00 (93.75, 831.50)	104.83 (22.75, 280.71)	0.003	0.251
%LAA of left lung (%)	3.23 (1.09, 37.41)	12.91 (4.26, 21.76)	0.216	12.00 (4.00, 22.00)	5.33 (1.14, 14.01)	0.007	0.406

*EID* Exercise-induced desatuation; *BMI* Body mass index; *6MWT* 6 min-walk test; *6MWD* 6 min-walk distance; *SpO*_*2rest*_ Resting blood oxygen saturation at the beginning of the test; *SpO*_*2min*_ Minimum blood oxygen saturation during the test; *SpO*_*2end*_ Blood oxygen saturation at the end of the test; *FVC* Forced vital capacity; *FVC%pred* Forced vital capacity as a percentage of predicted value; *FEV*_*1*_ Forced expiratory volume in the first second; *FEV*_*1*_*%pred* Forced expiratory volume in the first second as a percentage of predicted value; *D*_*L*_*CO SB* Lung carbon monoxide diffusion; *D*_*L*_*CO SB%pred* Lung carbon monoxide diffusion as a percentage of predicted value; *D*_*L*_*CO/VA* Lung carbon monoxide diffusion per liter of alveolar air volume; *D*_*L*_*CO/VA%pred* Lung carbon monoxide diffusion per liter of alveolar air volume as a percentage of predicted value; *mMRC* Modified Medical Research Council; *CAT* COPD Assessment Test; *PD15* 15th percentile point; *%LAA* % Low attenuation area

*P* * means the comparison between the new-EID and non-EID groups

### Correlation of EID with other outcome measures

With single regression analysis, the EID was significantly associated with 6MWD, D_L_CO SB% pred, D_L_CO/VA% pred, mMRC score, CAT score, CT-defined emphysema measurements, such as the mean density, PD15, emphysema volume and %LAA (Table 3). Only D_L_CO SB% pred, D_L_CO/VA% pred, CAT score, mean density, PD15, emphysema volume and %LAA were significant drivers of the EID in a multiple regression analysis (Table 3).

**Table 3:** Univariate and multiple logistic regression analysis of EID

Variables	EXP(B)	95%C.I	*P*	EXP(B)	95%C.I	*P*
gender	0.752	0.300–1.883	0.542			
Age (y)	1.011	0.976–1.047	0.559			
BMI (kg/m^2^)	0.951	0.862–1.100	0.282			
SpO_2rest_ (%)	1.069	0.907–1.260	0.428			
6MWD (m)	0.994	0.990–0.998	0.005	0.996	0.992–1.000	0.071
FVC% pred	0.999	0.983–1.016	0.914			
FEV1% pred	0.993	0.979–1.008	0.356			
D_L_CO SB% pred (%)	0.973	0.956 ~ 0.990	0.002	0.970	0.952–0.988	0.001
D_L_CO/VA% pred (%)	0.967	0.950–0.985	< 0.001	0.962	0.940–0.985	0.002
mMRC dyspnea scale	1.453	1.007–2.097	0.046	1.195	0.693–2.061	0.522
CAT score	1.121	1.055–1.3192	< 0.001	1.142	1.052–1.241	0.002
BODE index	1.193	0.999–1.424	0.051			
mean density (HU)	0.983	0.968–0.999	0.036	0.985	0.969–1.000	0.048
PD15 (HU)	0.982	0.967–0.997	0.016	0.983	0.968–0.999	0.033
emphysema volume (ml)	1.001	1.000–1.001	0.019	1.001	1.000–1.001	0.049
%LAA (%)	1.052	1.011–1.093	0.012	1.045	1.004–1.089	0.033

*EID* Exercise-induced desatuation; *BMI* Body mass index; *6MWD* 6 min-walk distance; *SpO*_*2rest*_ Resting blood oxygen saturation at the beginning of the test; *FVC% pred* Forced vital capacity as a percentage of predicted value; *FEV1% pred* Forced expiratory volume in the first second as a percentage of predicted value; *D*_*L*_*CO SB% pred* Lung carbon monoxide diffusion as a percentage of predicted value; *D*_*L*_*CO/VA% pred* Lung carbon monoxide diffusion per liter of alveolar air volume as a percentage of predicted value; *mMRC* Modified Medical Research Council; *CAT* COPD Assessment Test; *PD15* 15th percentile point; *%LAA* % low attenuation areas

### Different factors predict EID

**Fig. 2:**
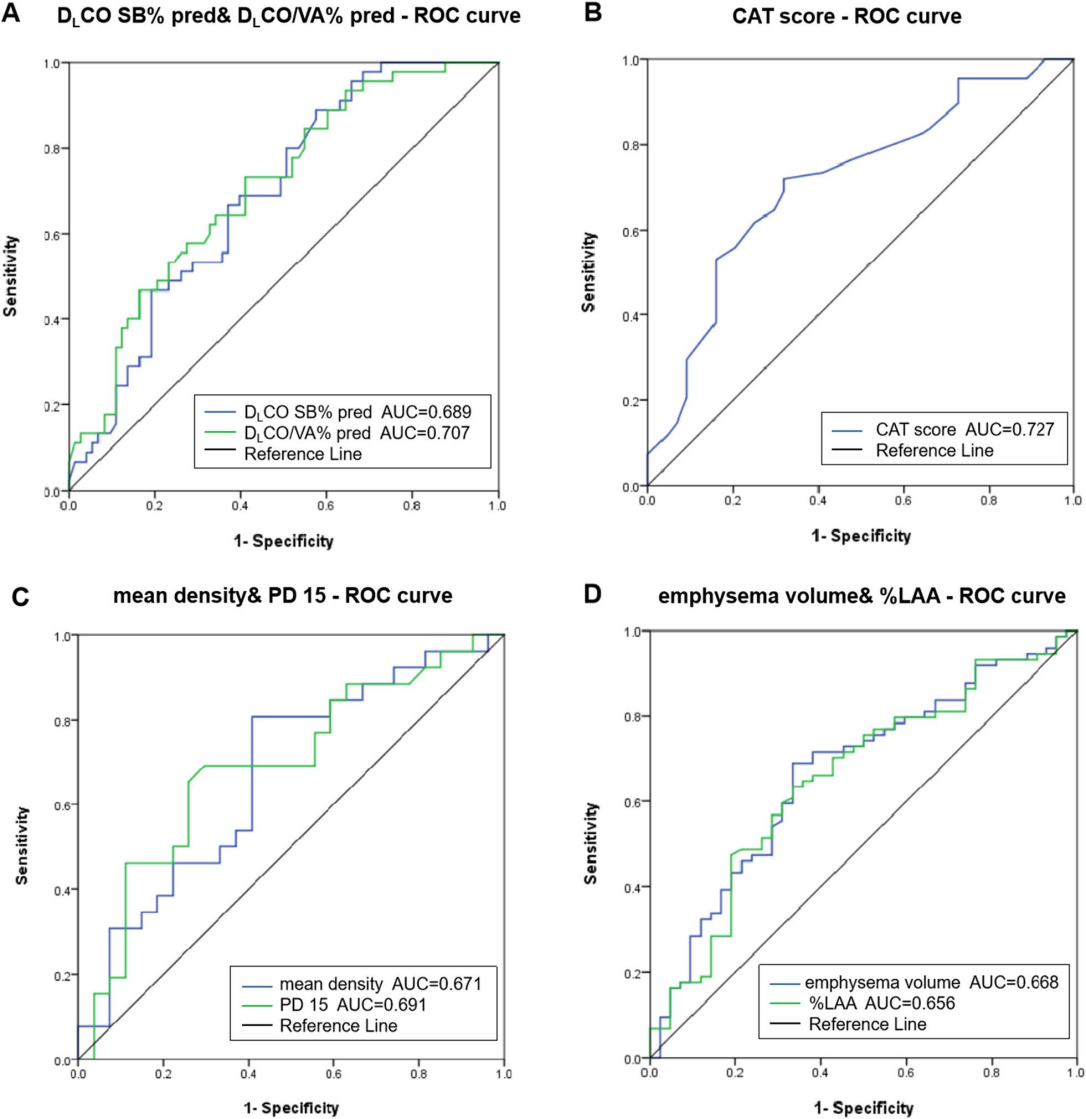
ROC curve analysis of the D_L_CO SB%pred, the D_L_CO/VA%pred (**A**), the CAT score (**B**), the mean density, the PD 15 (**C**), the emphysema volume, and the %LAA (**D**) for predicting EID. *ROC* receiver operating characteristic; *EID* exercise-induced desatuation; *D*_*L*_*CO SB%pred* lung carbon monoxide diffusion as a percentage of predicted value; *D*_*L*_*CO/VA%pred* lung carbon monoxide diffusion per liter of alveolar air volume as a percentage of predicted value; *CAT* COPD Assessment Test; *PD15* 15th percentile point; *%LAA* % low attenuation areas

In terms of diffusion function, the AUC of the D_L_CO SB percent pred predicting EID was 0.689, whereas the AUC of the D_L_CO/VA percent pred was 0.707, according to the ROC curve (Fig. 2A). The D_L_CO SB% pred and D_L_CO/VA% pred cutoff values were 50.45% and 80.9%, respectively (Table 4). The AUC of the CAT score predicting EID was 0.727 (Fig. 2B), and the cutoff value was 15 points, according to the ROC curve (Table 4). The AUCs for PD15, mean density, emphysema volume, and%LAA in CT-defined emphysema were 0.691, 0.671, 0.668, and 0.656 (Fig. 2C, D), respectively, for PD15, mean density, emphysema volume, and %LAA. − 955.00HU, − 856.46HU, 338.14 ml, and 7.63% were the cutoff values (Table 4).

**Table 4:** ROC curve analysis for predicting EID

Variables	AUC	Cut-off value	Sensitivity	Specificity	*P*
CAT score	0.727	15	0.753	0.66	< 0.001
D_L_CO SB%pred (%)	0.689	50.45	0.889	0.425	0.001
D_L_CO/VA%pred (%)	0.707	80.90	0.733	0.589	< 0.001
mean density (HU)	0.671	− 856.46	0.808	0.593	0.033
PD15 (HU)	0.691	− 955.00	0.692	0.704	0.017
emphysema volume (ml)	0.668	338.14	0.689	0.667	0.003
%LAA (%)	0.656	7.63%	0.635	0.667	0.005

*ROC* Receiver operating characteristic; *EID* Exercise-induced desatuation; *CAT* COPD Assessment Test; *AUC* Area under curve; *D*_*L*_*CO SB% pred* Lung carbon monoxide diffusion as a percentage of predicted value; *D*_*L*_*CO/VA% pred* Lung carbon monoxide diffusion per liter of alveolar air volume as a percentage of predicted value; *PD15* 15th percentile point; *%LAA* % Low attenuation areas

## Discussion

EID is prevalent in COPD patients and is a phenomenon that persists over time [9, 28]. Both the 2014 ERS/ATS and the 2021 Chinese version 6MWT guidelines require continuous oxygen saturation monitoring during exercise [23, 29]. The remarks about EID, on the other hand, said “drop 4% or SpO_2_ < 90%,” without indicating whether the decline was SpO_2rest_–SpO_2end_ or SpO_2rest_–SpO_2min_. The SpO_2end_ was shown to be similar to the SpO_2min_, but it did not reliably predict the SpO_2min_ in individuals who slowed down during exercise due to “exertion” or even rested to compensate for ventilation [30]. Chuang et al. discovered that patients who were desaturated and subsequently re-saturated had a higher FEV1/FVC ratio (*P* = 0.01) and reduced air retention [31]. A corresponding trend can be seen in our study too. Patients who were partially desaturated and re-saturated (n = 13) had lower blood oxygen variability (*P* < 0.001). They had improved diffusion, ventilation, and less emphysema when it came to lung function and CT defined emphysema. Thus, they may be more likely to return to non-hypoxic levels in a relatively “effortless” condition at the end of exercise. Although no statistical differences were observed for the appeal indices, a numerical trend may be seen (Table 1), and perhaps a larger sample size might yield an appreciable outcome. When compared the EID cohorts functional outcomes with those without EID, there were statistically significant variations in the SpO_2_ changes, 6MWD, and questionnaire scores (mMRC and CAT). However, the differences in pulmonary function and CT-defined emphysema were not statistically significant. This indicates that even while patients have not yet reached cutoff levels for lung function or CT-defined emphysema, clinicians still need to consider those with a more prominent “symptom burden” and reduced walking distance (which may be causally connected to EID).

After the pandemic, the following economic recession and shrank fiscal allotment, our local community hospitals can not afford Bluetooth real-time monitoring devices, as we learned from primary physician colleagues in National COPD network. Besides, most COPD patients we encountered in clinic were in low social-economic-status and 6MWT is not covered by health insurance in some low-income provinces of China. Many physicians executed the 6MWT by having the patient wear a portable finger clip oximeter to measure SpO_2_ throughout the test. Since it is against the guideline for the assessor to walk alongside the patient being evaluated, it is nearly impossible to accurately obtain SpO_2min_. In our study, Bluetooth real-time transmission technology was used so that SpO_2_ data could be sent to the iPad in real-time while the patient was being assessed. When the test is over, the assessor may immediately see changes in the patient's SpO_2_ over the course of the 6MWT. More EID events can be discovered by finding SpO_2min_ rather than collecting the difference between SpO_2rest_ and SpO_2end_. This also implies that by focusing on SpO_2min_, it is mostly possible to prevent misclassifying some desaturated-resaturated patients as “non-EID patients”, hence underestimating the risk of EID in this group. The rate of EID increased with GOLD classification in both methods, with the highest rate of EID at 6MWT in patients with pulmonary function stage IV, which is consistent with other national and international studies [6–10]. It’s worth mentioning that even in patients with stage I, the prevalence of EID was as high as 50% (12/24) in this study. Although the short sample size could be one of the causes contributing to the high prevalence, it also implies that patients with minor airflow restriction are at risk for EID and should be of concern to doctors.

It is worth noting that a high agreement between the two approaches was reported in our study (kappa = 0.807), although method A identified more patients with EID. This highlights the significance of continuous SpO_2_ measurement, which is the first choice for financially capable healthcare facilities. However, SpO_2rest_–SpO_2end_ measurements alone, without the aid of sophisticated continuous monitoring devices, can effectively detect EID in hospitals with limited resources, especially when assessing those without resting hypoxemia. This is practical and economical.

Several factors contribute to the formation of EID during exercise, including increased oxygen demand during exercise, dynamic lung hyperinflation, and ventilation-perfusion mismatch [32]. Intermittent hypoxia in patients during exercise may have the same long-term consequences as persistent hypoxemia. Patients with EID exhibited a 38 m loss in 6MWD at 1-year follow-up [33], as well as a faster rate of lung function decline, more frequent acute exacerbations [3, 34], more frequent acute exacerbations [3, 6] and a higher incidence of nocturnal hypoxia than those without EID [35]. As a result, prompt evaluation of the onset of EID and interventional treatment are critical components of conventional long-term COPD care.

Furthermore, the 6MWT guidelines consider SpO_2_ of less than 85% or 80% as a criterion for trial termination to limit the risk of malignant cardiac events or other adverse events [23, 29]. However, it has been suggested that SpO_2_ less than 80% in stable COPD patients undergoing 6MWT under the supervision of an experienced physical therapist is not associated with adverse events [36]. As a result, it’s thought that terminating the test due to a SpO_2_ of less than 80% in stable COPD patients isn’t necessary. When examining 6MWT in clinical practice, some COPD patients are likely to have tolerated hypoxia and believe they can complete the test despite a reduction in SpO_2_. Stopping the test due to a drop in SpO_2_ would not accurately reflect this group of patients' true exercise capacity. Therefore, it's critical to identify the clinical characteristics of COPD patients at risk of EID during the 6MWT so that this group of patients can be instructed to walk with oxygen and prevent having the test terminated due to hypoxia. It can reflect their true exercise capacity and lessen the risk of hypoxia-related complications. Furthermore, it aids in the early initiation of oxygen therapy prescriptions, thereby delaying the adverse prognosis induced by hypoxia.

Most studies consider diffusion function as a good predictor of EID. Patients with low D_L_CO SB% pred had a higher risk of EID with a threshold of 62%, according to Hadeli et al. [37] in a large cohort (n = 8000). The ECLIPSE cohort study showed that age, female, SpO_2rest_ ≤ 95%, D_L_CO SB% pred < 50%, and FEV1% pred < 45% had a high predictive value for EID [6, 9]. Both D_L_CO SB% pred and D_L_CO/VA% pred were found to be good predictors of EID in our study. Furthermore, we discovered that D_L_CO/VA% pred had somewhat superior predictive power (AUC = 0.707) than D_L_CO SB% pred (AUC = 0.689), despite the fact that few research have examined their predictive value for EID. D_L_CO SB% pred was marginally better than D_L_CO/VA% pred in predicting EID in a retrospective investigation of 97 patients with respiratory disease (58 with interstitial lung disease), with both having a threshold of 55% [38], contradicting to the findings of the current study. One of the explanations for the contradictory conclusions could be the various research populations primarily targeted in the current study compared to prior study. The leading indicator of diffusion function is usually D_L_CO SB% pred. However, when diffusion volume alveolar (VA) falls, D_L_CO SB% pred drops, therefore the VA is commonly used in the evaluation of diffusion function to rule out the effect of lung volume on diffusion volume, i.e. D_L_CO/VA. Under exercise, oxygen exchange is complicated, involving changes in pulmonary blood flow, lung capacity, and metabolism. D_L_CO/ VA% pred is a better predictor of EID than D_L_CO SB% pred owing to the fact that D_L_CO/VA% pred considers both intrinsic and volumetric diffusing capacity, making it a more comprehensive measure of diffusing capacity. In our investigation, the D_L_CO SB% pred threshold value for diagnosing EID was similar to the results of earlier studies. The threshold value for D_L_CO/VA% pred to diagnosis EID was 80.9%, indicating that included VA in the diffusing capacity study is more indicative of the likelihood of EID in COPD patients with mild diffusion impairment. It also emphasizes that in order to be more keenly aware of the likelihood of EID, doctors should pay attention to both D_L_CO SB% pred and D_L_CO/VA% pred declines in pulmonary function indices.

In this study, patients with EID in 6MWT had significantly higher mMRC scores, CAT scores, and BODE indexes than non-EID patients. It suggests that individuals with EID had more severe dyspnea, a higher symptom burden, and a worse illness outcome than patients without EID. Surprisingly, the CAT score had a stronger predictive value for EID than the other two questionnaire scores (AUC = 0.727). Within 2–3 min, the CAT score measured health-related quality of life and illness symptom load. For patients who are unable to complete the 6MWT or conduct continuous SpO_2_ measurements; who do not have access to a diffusion function test in primary care settings; or who live in low-income areas, the CAT score may be used to screen a subset of patients at high risk of developing EID. It is easy to perform, time-consuming, and has no staffing or site requirements, so it can be an option for primary and community care units as a more convenient and rapid method to assess patients with risk of EID.

CT-defined emphysema is a useful complement to subjective visual assessment. It was found significantly correlated with D_L_CO SB% pred, D_L_CO/VA% pred and FEV1% pred [17, 39]. PD15 is also the most significantly connected with D_L_CO/VA% pred in diffusion function, followed by %LAA [39], which explains the strong correlation of these two indicators with EID in multifactorial regression analysis. EID initiation and development are determined by the degree of emphysema [18], and investigations have demonstrated that %LAA is independently associated with EID [16, 17]. According to Marie Waatevik et al. the median %LAA in patients with COPD who formed EID in 6MWT for the first time was 12.6%, and the median %LAA in patients with COPD who developed EID in 6MWT for the second time was 21.7% [18]. In our study, the median %LAA of COPD patients who developed EID in the 6MWT was 11%, and the cut-off value for predicting EID was 7.63%. Many COPD patients who acquired EID only had a low %LAA, according to research. The lower degree of airflow limitation (higher FEV1% pred) in our population may have resulted in a relatively low %LAA, implying that even mild to moderate emphysema (%LAA 5–25%) [10] is enough to impact the development of EID. In our study, we found a more excellent value of PD15 (AUC = 0.691) than %LAA (AUC = 0.656) in predicting EID, which may be due to the fact that PD15 is influenced not only by the volume of emphysema but also by the amount of residual lung tissue available for gas exchange [39] and is more stable than %LAA in assessing the extent of emphysema [40]. Moreover, PD15 also diminishes with age, making it a stronger indicator of the existence and progression of emphysema [15].

Despite the fact that CT is becoming more widespread in modern medical practice, this does not indicate that all patients with COPD should have a CT scan to determine the amount of emphysema and the risk of EID; after all, CT is still an expensive and invasive procedure. However, for COPD patients who have undergone CT either as part of disease surveillance or for other comorbidities, the use of imaging information from CT to effectively assess all and localized pulmonary changes in a short period and to accurately quantify the risk of emphysema can predict the risk of EID, which can guide future individualized treatment and prognostic assessment of patients with COPD. This has implications for COPD patients’ future individualized treatment and prognostic evaluation.

There are several limitations to this study as well. First, the study's sample size was tiny, and the data came from a single center. Second, we did not analyse cardiopulmonary comorbidities in our patients. Atrial fibrillation and hypertension have been linked to lower SpO_2_ during the 6MWT in some studies [6, 8]. It’s unclear whether comorbidities were present in the study's participants, which could affect the accuracy of the findings. Third, several factors influence CT-defined emphysema, including the thickness of the scan layer, the degree of obesity, and the depth of deep inspiration [41]. Other research has found that when utilizing the threshold approach to measure emphysema, the degree of emphysema increases as the layer thickness gets thinner. This pattern reduces as the threshold is raised [42], whereas, 37 individuals were scanned with a 5-mm layer thickness in the current study, which may have influenced the CT quantification results.

## Conclusions

This study emphasizes the need of continuous SpO_2_ monitoring to determine SpO_2min_ during the 6MWT in order to capture more EID in COPD patients. Standardized criteria for EID assessment and determination should be identified for in-depth investigation in future clinical trial studies. When conditions are limited, it is also sufficient to assess EID in COPD patients by measuring SpO_2rest_–SpO_2end_ ≥ 4% and/or SpO_2end_ < 90%. In terms of predictors of EID, D_L_CO SB% pred and D_L_CO/VA% pred, as well as CAT score and CT-defined emphysema, are all statistically significant test factors for determining EID. The above indicators were used to find COPD patients with a high risk of EID, who were advised to take oxygen in 6MWT to avoid the exercise termination due to hypoxia and to reflect the best exercise capacity; meanwhile, early intervention treatment was performed to improve patient prognosis by identifying the risk of developing EID in these patients.

## Data Availability

The data that support the findings of this study are available from Dr. Ting Yang. These data were used under license for the current study, so these data are not publicly available. However, the data are available from the authors upon reasonable request and with permission from corresponding authors.

## Abbreviations

6MWT: 6 Minute-walk test
6MWD: 6 Minute-walk distance
%LAA: % Low attenuation areas
AUC: Area under the curve
BMI: Body mass index
COPD: Chronic obstructive pulmonary disease
CAT: COPD assessment test
D_L_CO SB: Lung carbon monoxide diffusion
D_L_CO SB% pred: Lung carbon monoxide diffusion as a percentage of predicted value
D_L_CO/VA: Lung carbon monoxide diffusion per liter of alveolar air volume
D_L_CO/VA% pred: Lung carbon monoxide diffusion per liter of alveolar air volume as a percentage of predicted value
EID: Exercise-induced desatuation
FVC: Forced vital capacity
FVC% pred: Forced vital capacity as a percentage of predicted value
FEV1: Forced expiratory volume in the first second
FEV1% pred: Forced expiratory volume in the first second as a percentage of predicted value
HU: Hounsfield units
mMRC: Modified Medical Research Council
PD15: 15Th percentile point
ROC: Receiver operating characteristic
SpO_2_: Blood oxygen saturation
SpO_2rest_: Resting blood oxygen saturation at the beginning of the test
SpO_2min_: Minimum blood oxygen saturation during the test
SpO_2end_: Blood oxygen saturation at the end of the test
VA: Alveolar volume
